# Le corticosurrénalome: une cause exceptionnelle d’hyperaldostéronisme primaire

**DOI:** 10.11604/pamj.2018.31.60.16973

**Published:** 2018-09-27

**Authors:** Baha Zantour, Ines Charrada, Zohra Elati, Fatma Larbi Ammari, Fadia Boubaker, Sondes Arfa, Olfa Berriche, Wafa Alaya, Mohamed Habib Sfar

**Affiliations:** 1 Service d’Endocrinologie et Médecine Interne, Hôpital Tahar Sfar, Mahdia, Tunisie

**Keywords:** Corticosurrénalome malin, hyperaldostéronisme primaire, surrénale, Malignant corticosurrenaloma, primary hyperaldosteronism, adrenal

## Abstract

Le corticosurrénalome est une tumeur maligne rare de la cortico-surrénale. Il secrète souvent des corticostéroïdes, des stéroïdes sexuels et des précurseurs. Le corticosurrénalome producteur d'aldostérone est très rare, 1cas/10 millions d'habitants. Nous rapportons l'observation d'un homme de 38 ans se présentant pour hypertension artérielle sévère associée à une hypokaliémie profonde (2.2 mmol/l). L'exploration a conclu à un hyperaldostéronisme primaire (aldostérone = 2645pmol/l, rapport aldostérone/rénine = 327pmol/mUI), avec hypersécrétion de glucocorticoïdes. Le scanner abdomino-pelvien a montré une masse surrénalienne gauche de 9cm, mal limitée et hétérogène, infiltrant la graisse autour et le diaphragme, envahissant la veine rénale gauche, avec adénopathie régionale et nodule hépatique de 4 cm. Le patient a eu une néphrectomie élargie, suivie d'une hépatectomie droite deux mois après entrainant une rémission. Un an après le patient a développé des métastases pulmonaires. Nous concluons que l'hyperaldostéronisme primaire peut être un mode de révélation du corticosurrénalome, on devra y penser malgré son caractère exceptionnel.

## Abstract (en)

Corticosurrenaloma is a rare malignant tumor of the adrenal gland that often secretes corticosteroids, sex steroids and precursors. Aldosterone-producing corticosurrenaloma is very rare, accounting for 1 case/10million inhabitants. We report the case of a 38-year old man presenting with severe arterial hypertension associated with deep hypokalaemia (2.2 mmol/L). Exploration showed primary hyperaldosteronism (aldosterone = 2645 pmol/l, aldosterone/renin ratio = 327 pmol/MUI), with hypersecretion of glucocorticoids. Abdominopelvic CT scan revealed left poorly differentiated and heterogeneous adrenal mass measuring 9cm, infiltrating into the surrounding adipose tissue and the diaphragm, extending into the left renal vein, with regional adenopathy and hepatic nodule measuring 4cm. The patient underwent radical nephrectomy followed by right hepatectomy two months after resulting in remission. A year after the patient developed lung metastases. This study highlights that corticosurrenaloma should be suspected in patients with primary aldosteronism despite its rarity.

## Introduction

Le corticosurrénalome (CS) est une tumeur endocrine maligne rare de la corticosurrénale. Son incidence est d'environ 1 à deux nouveaux cas par million et par an [1]. Le plus fréquemment, le diagnostic est posé devant des signes d'hypersécrétion hormonale dont la symptomatologie est variable, dépendant de la voie de synthèse hormonale développée par le tissu endocrinien tumoral et/ou devant un syndrome tumoral. Dans tous les cas, une altération de l'état général est très souvent notée et le mode de révélation est très rapide [2]. Le CS producteur d'aldostérone est très rare, un cas par dix millions d'habitants [3]. Nous rapportons ici un cas de CS secrétant de l'aldostérone et des gluco-corticoïdes, découvert devant une HTA et une hypokaliémie sévères isolées, sans altération de l'état général ni de syndrome tumoral ni de signes cliniques en faveur de la sécrétion gluco-corticoide.

## Patient et observation

Il s'agit d'un homme âgé de 37 ans, commerçant, marié, père d'une fille, aux antécédents familiaux d'hypertension artérielle chez la mère. Son histoire remonte à un mois auparavant, soit le 13/01/17 marquée par la survenue de lipothymie amenant le malade à consulter les urgences où une pression artérielle à 190/85 mm Hg a été objectivée. Il a été mis initialement sous Nicardipine LP 50mg deux fois par jour. A l'interrogatoire, le patient rapportait des céphalées, des crampes musculaires, une hypersudation et une prise de poids. A l'examen clinique, Il n'avait pas de vergetures ni d'œdèmes des membres inférieurs, poids 78kg, taille 1.65m, indice de masse corporelle 28,67 kg/m^2^. Le bilan biologique a révélé une hypokaliémie profonde à 2,2 mmol/l, hyperkaliurèse à 98,8 mmol/l, TSH 1.44 mUI/l, protéinurie de 24h négative, calcémie: 2,42 mmol/l. Le reste du bilan a montré: clairance de la créatinine 74 ml/min, glycémie 5,8 mmol/l, cholestérol 6,5 mmol/l, triglycérides 1,38 mmol/l, HDL cholestérol 1,53 mmol/l et LDL cholestérol 4,3 mmol/l. L'exploration hormonale en conditions standardisées [4] (patient en position assise depuis 15 min et après 2 heures de réveil) a montré: Aldostéronémie 2645 pmol/l, Rénine 8,1 mUI/l, Rapport aldostérone/rénine 327 pmol/mUI. L'HAP a été ainsi confirmé devant une aldostéronémie largement supérieure au seuil de 550 pmol/l, une rénine basse < 5 mUI/l et un rapport supérieur à 64. L'échographie abdominale a montré une masse surrénalienne gauche finement calcifiée dépassant 4 cm de grand axe, un nodule hypoéchogène du segment VIII du foie de 36 mm de grand axe, des micro lithiases rénales bilatérales sans retentissement.

**Figure 1:**
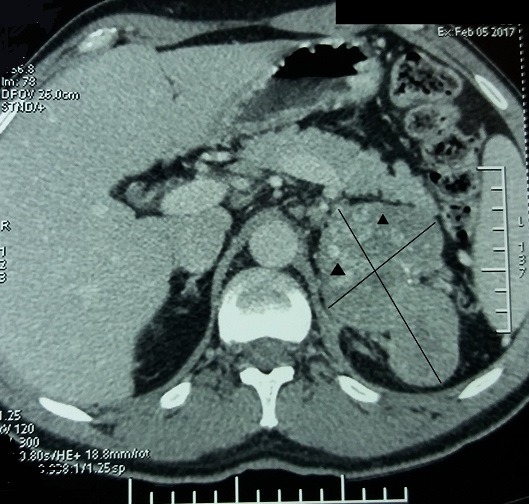
TDM abdominale: coupe transversale: corticosurrénalome gauche de 9 cm; les flèches montrent les calcifications et les nécroses

**Figure 2:**
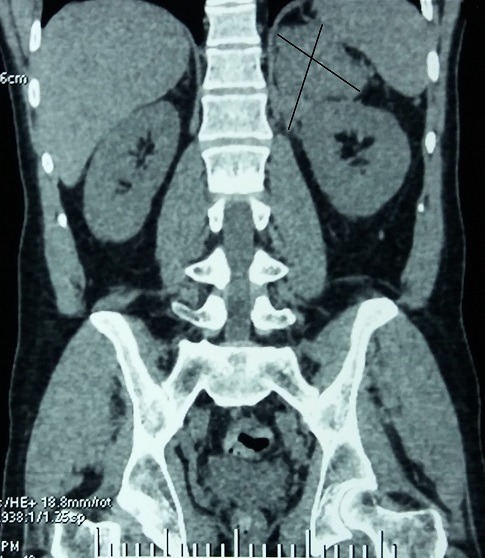
TDM abdominale: coupe sagittale de la tumeur

**Figure 3:**
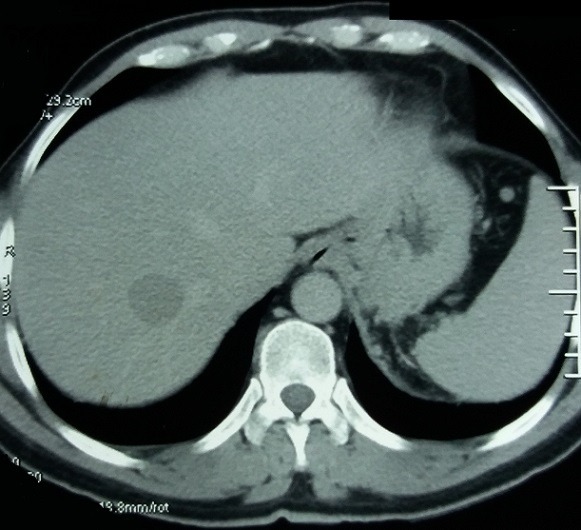
TDM abdominale: métastase hépatique droite de 4 cm

Le scanner abdomino-pelvien fait le 05/02/17 a montré une masse surrénalienne gauche de 9cm, mal limitée et hétérogène avec hémorragie, nécrose et calcifications, infiltration de la graisse autour et du diaphragme, invasion de la veine rénale gauche sans extension à la veine cave, adénopathie régionale (Figure 1, Figure 2), et nodule secondaire hépatique de 4cm (Figure 3). Cet aspect était en faveur d'un corticosurrénalome stade IV de l'ENSAT [5] nécessitant la poursuite des explorations par un bilan hormonal et d'extension Explorations hormonales: cortisolémie 8h 175,8 µg/l (> 130 µg/l), cortisolémie 20h 188,6 µg/l (> 75 µg/l), cortisol libre urinaire CLU 192 µg/24h (> 80µg/24H). L'élévation du cortisol plasmatique et urinaire et l'inversion du cycle du cortisol étaient très en faveur d'un hypercorticisme qui a été confirmé par l'absence de freinage au test de freinage faible par la dexamethazone (2mg/jour pendant 2 jours): Cortisolémie: 185,4 microg/l, CLU: 142.5 microg/24h. Le dosage de l'ACTH a trouvé une valeur freinée à 3,9 pg/ml. Il s'agissait donc d'un syndrome de Cushing ACTH indépendant. Il n'y avait pas d'excès de production des stéroïdes sexuels, œstradiol: 25 ng/ml, testostérone plasmatique: 2.52 ng/ml. Le dosage des dérivés méthoxylés urinaires des 24h était normal. A l'issue de cet ensemble d'investigations convergentes, le diagnostic retenu est celui d'un CS stade IV avec invasion loco-régionale, métastase hépatique, et extension à la veine rénale gauche, hypersécrétant d'aldostérone et de glucocorticoïdes. Le patient a été mis sous spironolactone 100 mg un comprimé par jour et chlorure de potassium 600 mg, un comprimé trois fois par jour. Il a eu une néphrectomie élargie, suivie d'une hépatectomie droite deux mois après entrainant une rémission. Un an après, soit en mars 2018, le patient a développé des métastases pulmonaires qui ont été traitées par radiothérapie externe. Le test au Tetracosactide (ACTH synthétique) 1 microg fait en mai 2018 a montré une insuffisance surrénalienne. Le patient est actuellement sous hydrocortisone 10 mg un comprimé par jour, sa pression artérielle et kaliémie sont normales, a perdu 10kg de poids. Un traitement par mitotane est prévu.

## Discussion

Le CS est une tumeur maligne rare dont le tableau clinique est variable. Les CS fonctionnels ou sécrétants entraînent un syndrome endocrinien. Par ordre de fréquence, il peut s'agir d'un hypercorticisme isolé ou associé à une virilisation ou une féminisation [1, 6], d'une hyperandrogénie pouvant provoquer chez le nouveau-né une ambiguité sexuelle à la naissance [7] et exceptionnellement une hyperoestrogénie ou un HAP [8], c'est le cas de notre patient. L'HAP peut être un mode de révélation d'un CS [9]. Il est responsable d'un tableau clinico-biologique dont la sévérité peut être en rapport avec la nature carcinomateuse de la lésion. La littérature confirme la rareté de l'exclusivité de la production d'aldostérone par le CS [9]. La TDM abdominale est l'examen de première intention pour l'exploration des surrénales devant un syndrome d'hyper-sécrétion hormonale [4]. L'existence d'une hémorragie intra-tumorale ou d'une nécrose donne un aspect scannographique hétérogène en faveur de la malignité sans que ce soit spécifique [10]. Les adénomes de Conn sont souvent inférieurs à 5cm, tandis que les carcinomes sont souvent plus larges [3]. A notre connaissance, 64 cas de CS entrainant un HAP ont été rapportés [7, 9, 11-15]. Seccia TM et al. ont fait une revue de 58 cas de CS produisant l'aldostérone [2]. Selon cette revue, l'hypokaliémie était présente dans tous les cas sauf un seul cas [8], l'hypertension artérielle a été trouvée dans tous les cas sauf 5 cas [2, 12]. Plusieurs hypothèses ont été avancées pour expliquer l'absence d'hypertension artérielle, mais le mécanisme exact est inconnu. Dans 10% des cas il y avait une augmentation modérée du cortisol associée et la S-DHEA était souvent normale [2]. Les taux des 17-hydroxystéroides et des 17-cétosteroides, mesurés dans 28 cas, étaient augmentés dans 8% et 6% des cas respectivement.

Le traitement de première intention des CS quel que soit le type de sécrétion est la chirurgie résultant en une rémission dans 0 à 50% des cas selon le stade lors de l'intervention [16]. La survie moyenne est de 4 à 8 mois lorsque les localisations secondaires existent [17]. Chez notre patient, la chirurgie a entrainé une rémission de un an avec normalisation de la pression artérielle et de la kaliémie et insuffisance cortisolique malgré la présence de métastase hépatique et d'une extension à la veine rénale. La chirurgie surrénalienne doit être proposée systématiquement dans les CS même en présence de métastases si une résection complète peut être obtenue [18]. De plus, la chirurgie des métastases sera envisagée, en cas de facteurs pronostiques favorables, lorsque la résection peut être complète et à chaque fois qu'une réponse tumorale à la chimiothérapie est observée [5]. Chez notre patient, la chirurgie surrénalienne et hépatique ont pu allonger la survie et permis de maitriser l'hyper-sécrétion hormonale. L'alternative aux ré-interventions reste le mitotane et les polychimiothérapies. En cas de récidive où l'exérèse a été incomplète ou récusée, le traitement de première ligne est le mitotane. Le traitement par mitotane doit être adapté au taux sérique qui doit être supérieur à 14 μg/ml pour atteindre une dose thérapeutique efficace [14]. Le mitotanne associé à la chimiothérapie est plus efficace que le mitotane seul. L'association mitotane/étoposide/doxorubicine/cisplatine a été démontrée supérieure à l'association mitotane/streptozotocine dans les formes non résécables et/ou métastatiques dans l'étude FIRM-ACT [19]. La radiothérapie adjuvante du lit tumoral n'a pas d'effet favorable démontré. Par contre, la radiothérapie hépatique, pulmonaire ou osseuse peut être utilisée notamment sur les localisations hépatiques et pulmonaires lorsque celles-ci sont peu nombreuses et d'agressivité intermédiaire [19]. Une radiothérapie thoracique des métastases pulmonaires a été pratiquée chez notre patient, avec un recul actuel de 6 mois.

## Conclusion

Le CS est une tumeur maligne rare. Le diagnostic a été établi dans notre cas sur des critères cliniques, biologiques et radiologiques malgré le caractère exceptionnel et trompeur de son mode de révélation qui était l'hyperaldostéronisme primaire. La prise en charge nécessite une collaboration multidisciplinaire qui a été mise en œuvre pour notre patient. L'évolution est imprévisible. Chez notre patient, la chirurgie de la tumeur primitive et de la métastase hépatique puis la radiothérapie des métastases pulmonaires a permis d'allonger la survie et de traiter efficacement l'hyper-sécrétion hormonale. L'étude génétique de la pièce tumorale qui a manqué dans notre observation aurait eu l'intérêt d'estimer le pronostic et l'évolution.

## Conflits d’intérêts

Les auteurs ne déclarent aucun conflit d'intérêts.

## Contributions des auteurs

Chacun des auteurs a participé à la prise en charge du patient du diagnostic au traitement. Les 2 premiers auteurs ont réalisé la rédaction de l'article. Tous les auteurs ont lu et approuvé la version finale du manuscrit.
